# Impact of the Pandemic on STEAM Disciplines in the Sixth Grade of Primary Education

**DOI:** 10.3390/ejihpe12080071

**Published:** 2022-08-04

**Authors:** Pablo Dúo-Terrón, Francisco-Javier Hinojo-Lucena, Antonio-José Moreno-Guerrero, Jesús López-Belmonte

**Affiliations:** Department of Didactics and School Organisation, University of Granada, 51001 Ceuta, Spain

**Keywords:** pandemic, STEAM, computational thinking, makerspaces, future classroom

## Abstract

The demand for professionals entering the labor market requires knowledge and disciplines in the areas of Science, Technology, Engineering, Art and Mathematics (STEAM). Schools are the first link to train competent students for today’s society. However, the pandemic has conditioned the teaching–learning methodologies based on promoting STEAM in educational centers, which is the reason that leads us to carry out this study. The main objective of the research is to evaluate the STEAM dimensions in the sixth grade of primary education in times of pandemic. The study method is based on a quasi-experimental, descriptive and correlational design with an experimental group and a control group. The data are collected through a validated questionnaire, pre-test and post-test, which develops an assessment of student collaboration in STEAM activities. The sample is made up of 142 Spanish students, of which 68 belong to the control group and 74 to the experimental group. The conclusions of the study highlight that the active methodologies, based on computational thinking and on makerspaces of the future classroom, influenced the STEAM dimensions of the experimental group before the pandemic. However, the pandemic and the health restrictions in face-to-face classes led to a negative assessment of the experimental group in the STEAM dimensions.

## 1. Introduction

Information and communication technologies (ICT) in education are used more frequently as an online communication and collaboration channel between teachers and students; however, after COVID-19, their use has accelerated. Even Ref [1] points to this era as the fourth industrial revolution, where it is necessary to cultivate creative convergence talents that can deal with uncertainty and coexist with technology. Teachers and students have adapted to new learning with social distancing, and the demand for new teaching methods is growing in the educational field [2]. Social changes and technological advances impose changes in the teaching and learning models. There is a factual need to incorporate new methodological approaches [3] that enhance creativity, the ability to solve problems and the development of new digital skills in students [4,5]. In this sense, ICTs stimulate positive responses in students and favor their attention toward the areas of Science, Technology, Engineering and Mathematics (STEM) [6,7], especially when they work in a transversal and integrated way at the curricular level [8].

STEAM approaches incorporate the (A) of arts and creativity in education in the term STEM [9,10]; in this way, learning is more integrated, meaningful and attractive for students [11]. It is a relatively new construction in education [12] and can be improved through specific educational programs [13]. STEAM increases interest in pedagogical approaches that can bring new methods for innovation in society [14]. STEAM in education (STEAM-EDU) allows cultivating talented students and meeting the demand for professions in today’s society [15] linked to the use of technological devices and tools, such as engineers, programmers, computer scientists or project leaders [3,16]. For this reason, the educational field must be the axis and the base of the world economic future in view of the emerging skills needs of the labor market [17] and must adopt anticipatory strategies in education [18]. However, there is little research on STEAM-EDU teaching methodologies and resources among teachers [19,20].

### 1.1. STEAM Disciplines in Education

This study investigates different STEAM-EDU disciplines and areas, including multidisciplinarity, interdisciplinarity and transdisciplinarity [21,22,23]. These disciplines have an impact on skills, such as linguistics or mathematics [24,25]. In addition, notable effects on the learning attitude, motivation, confidence, student success and relationships between students and teachers are evident [26,27,28]. STEAM-EDU confirms that a student can conceive of more than one discipline occupying the same school hours and curricular space [29].

Other STEAM-EDU-related disciplines within the scientific literature are peer interaction and problem solving, in which STEAM-EDU has a dominant influence [30]. In this sense, social development and project or team work enable real-world learning through inquiry, collaboration and positive communication in the classroom [31,32,33,34]. Critical thinking plays an important role in this process [31,35], which allows students to interact and self-assess using digital technology. Learning is facilitated when all students, more or less experienced, organize their work in a way that allows all participants the opportunity to see, discuss and engage in problem solving and shared practice [36].

One way to develop these STEAM-EDU skills are teaching methodologies aimed at learning based on problems, projects and collaborative, experiential and playful learning [37]. Physical spaces, learning and emotional elements also influence academic results in schools [1]. However, the impact of collaboration and communication skills is still low [30]. To this fact, it must be added that in recent years, the relationship and interaction between pairs of students as a result of the COVID-19 pandemic have caused educational systems to accelerate the use of technologies to relate, inserting the R of reflective learning [11], that is, STREAM. In addition, this method merges reading, writing and arts to connect the four STEM disciplines [37]. Students have a positive perception of digital platforms due to the availability of comments and quick access to learning materials [38].

### 1.2. STEAM-EDU and Computational Thinking

STEAM-EDU develops computational thinking skills at an early age [39], understood as the ability to formulate and represent problems to solve them through the use of tools, concepts and practices of the computer science discipline, such as abstraction, decomposition or use of simulations [40]. There are multiple fields that allow STEAM-EDU to work through computational thinking, such as robotics [41,42], block programing or artificial intelligence, which favor student problem solving [43,44] with benefits for the development of creative thinking [45,46]. Computational thinking “is acquiring great importance due to the evolution of new technologies, thus creating a global trend that considers programming in the classroom as a fundamental activity of the present and the future” [47] (p. 45). In addition, “it is currently considered one of the most demanded skills and, hence, its approach in the educational context” [48] (p. 1).

This fact has not gone unnoticed in the Spanish educational policy, which has published at the beginning of the year 2022 a new curriculum and included computational thinking in the Royal Decree 157/2022 of Primary Education [49], specifically in the area of knowledge of the medium, as a method to “decompose a problem into simpler parts, pattern recognition, model making, selection of relevant information and the creation of algorithms to automate processes of daily life” [49] (p. 24415). It also introduced computational thinking into the area of mathematics as one of the key skills in the future of students (“this thinking should be specifically trained and developed with guided methodologies and strategies” [49] (p. 24488)). The use of information technology and computer programs in the areas of education could be an effective movement to awaken vocations in young people who are more deeply involved in engineering, modeling, robotics, programing skills, artificial intelligence [50,51]. However, computational thinking should be understood as a support or methodological means for students to learn curricular content from different areas or subjects while learning digital skills [52].

Currently, computational thinking in schools is imperative for innovation but with knowledge and skills among educators and professionals [53]. Teachers should address student interest generated by STEAM by reference to prior knowledge and use collaborative tools, tutorials, materials and methods that mimic the real world through meaningful learning [54,55], that is, “know how”. However, it is necessary that the teachers themselves possess knowledge and skills in the use of technological resources and active methodologies [56,57].

### 1.3. Makerspaces and Future Classroom

The experimental group present in this research integrates makerspaces into their educational projects, that is, spaces to encourage creativity and collaborative learning through STEAM-EDU [58,59]. These spaces prompt students to improve their knowledge using collaborative learning and technology, unlike the traditional method [60]. In these spaces, students imagine, explore, experiment, test, manipulate, discuss and speculate [45]; these actions favor learning through projects and discovery. In addition, the use of STEAM-EDU together with ICT tools is useful to combat school failure, attend to diversity, encourage reading and promote inclusion [61,62]. Active methodologies together with adapted spaces provide meaningful learning that increases student motivation and participation, facilitating the acquisition of content and knowledge in a gamified and playful way [63].

**Figure 1:**
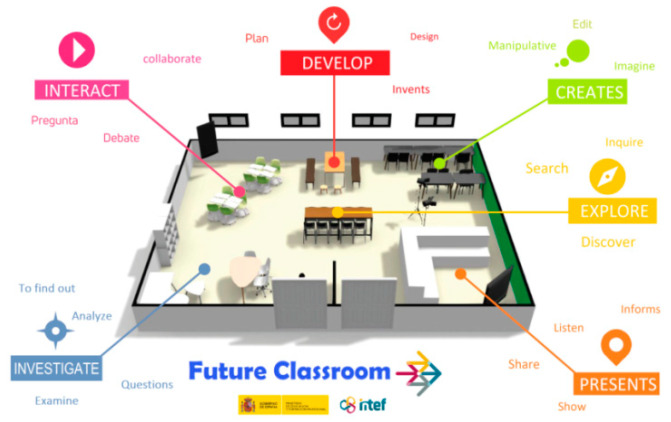
Spaces of the future classroom. INTEF.

From the European Schoolnet in Brussels, these makerspaces, called the Future Classroom Lab, have been promoted since 2012 with the aim of inspiring practicing teachers to rethink education and adapt their spaces in order to understand how they can positively impact student performance [64]. In Spain, the National Institute of Educational Technologies and Teacher Training (INTEF) and the different departments of education implement Classrooms of the Future (Figure 1) to provide support and training to teachers and educational centers. According to INTEF [65], learning spaces terms are: research, interact, explore, develop, create and present. The rise of makerspaces means that schools themselves are integrating these spaces into their educational plans to share resources and knowledge among students, motivating them to be thinkers, creatives and critics, as well as problem solvers, excellent communicators and collaborators [66]. The centers take into account the organization of learning spaces, the socio-emotional skills required for teamwork and projects, programing and designing activities [67]. Additionally, the spaces allow students to build, invent and establish relationships with all kinds of tools and utensils, such as 3D printers [6].

In the decade of the digital age and STEAM labor demands, organizations must adapt policies and infrastructure that allow continuous improvements of educational processes [3]. As STEAM-EDU is gaining popularity in K-12 schools, student assessment is needed to identify the most influential dimensions [46]. This reason leads us to the present study on peer interaction, collaboration, communication, research and transdisciplinary thinking in problem solving offered by STEAM after the emergence of COVID-19.

### 1.4. Justification and Objectives of the Study

The pandemic situation due to COVID-19 in March 2020 motivated all countries to adopt extreme measures to prevent the spread of the virus. In a few weeks, the educational systems changed and adapted their methodologies to the health regulations [68]; even face-to-face classes were suspended. In the Spanish context, schools resumed face-to-face classes with prevention, hygiene and health promotion measures against COVID-19 imposed by the Spanish Ministry of Education and Health [69]. Among the most outstanding measures was the decrease in student ratio, i.e., no more than 15 students per classroom. The use of masks was also mandatory, and the use of the center’s dependencies, meetings and assemblies between teachers and students was prohibited, and the minimum distance was 1.5 m between the students. They could also not share school supplies. In addition, there were 14-day lockdowns on students in the event they tested COVID+ or had close contact with a positive person.

Taking the theoretical framework as a reference, the possibility of carrying out cooperative activities among students was limited to digital platforms, videoconference applications, email. In this way, the students lost the possibility of fully benefiting from all the advantages of collaborative learning and experimentation. In addition, it caused a disadvantage in unfavorable sociodemographic environments [70]. This fact has generated in the educational field the need related to the use, learning and mastery of ICT [71]. Interdisciplinary projects with STEAM methodology after the pandemic can create a global and real vision of the knowledge that can be imparted in teaching [72]. In this sense, the COVID-19 pandemic must be used as an opportunity [70]. This reason leads us to investigate how the STEAM disciplines have been affected.

As a result of this situation, this research arises to expand knowledge about the impact that the pandemic has had on the STEAM-EDU disciplines in the primary education stage. We analyze and compare the different STEAM-EDU disciplines between a control group and an experimental group, the latter integrating computational thinking and the Classroom of the Future into its educational plans.

The main objective of this research is to evaluate the STEAM dimensions in the sixth grade of primary education in times of pandemic. There are five STEAM dimensions under study: Peer Interaction (PEER_INT), Positive Communication (POST_COM), Multiple Options (MUL_PATHS), Focus and Tasks (AA_TASK), Transversal Thinking (TRANS_THINK).

In addition, this general objective leads to the formulation of the following specific objectives:

- Compare how working with STEAM-EDU influences each of the dimensions of the study before the pandemic using different methodologies.
- Know the impact that the COVID-19 pandemic has had on STEAM disciplines.

Based on these objectives, the following hypotheses arise:

- H1: STEAM-EDU disciplines before the pandemic are more effective with an active methodology based on computational thinking and with makerspaces compared to a traditional methodology.
- H2: STEAM-EDU disciplines during the pandemic are better valued in the experimental group compared to the control group.

## 2. Materials and Methods

This research is a study that responds to a quasi-experimental design, with experimental and control groups and descriptive and correlational pre-test and post-test measurements, supported by a quantitative method in data treatment [73]. In order to proceed to a correct performance of the investigation, the study had the considerations of experts in these types of methodologies [74]. In addition, the analysis structure of previously reported impact database studies was followed to carry out a science-validated method [75].

**Figure 2:**
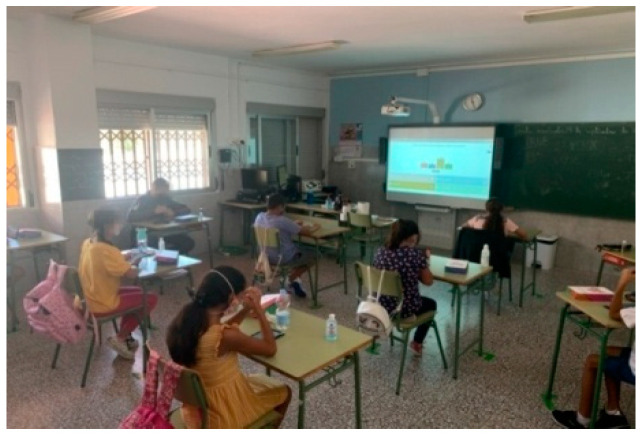
Post-pandemic experimental group.

**Figure 3:**
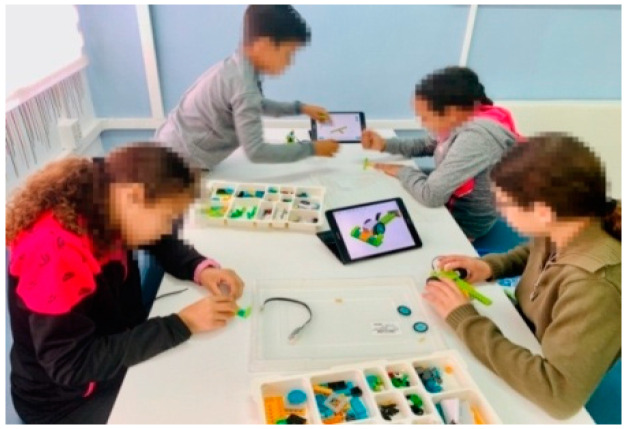
Pre-pandemic experimental group.

Specifically, during the research process in times of pandemic, the methodologies carried out by both the control and experimental groups were similar to the traditional one due to the restrictions on collaborative work and distancing imposed by the government of Spain (Figure 2). However, the experimental group before the pandemic used active methodologies based on computational thinking and makerspaces (Figure 3), while the control group used a traditional methodology.

### 2.1. Participants

In this study, the final sample is made up of 142 Spanish students, of which 68 belong to the control group and 74 to the experimental group. Six students from the control group and two students from the experimental group were excluded because they did not take the pre-test, the post-test or any of them. The educational center is publicly owned in the city of Ceuta, a Spanish city located in the north of the African continent and with more than 40% of the inhabitants of the Islamic religion [76]. This educational center was selected for including within its educational center project active methodologies based on computational thinking and having Classrooms of the Future with makerspaces, as indicated by the INTEF in its network map of centers in Spain [77]. The control group of students also belongs to the same educational level, the sixth grade of primary education. It was selected because it was located in the same area of influence and constituency as the center of the experimental group in the city of Ceuta, that is, with the same sociodemographic characteristics to avoid bias. The previous analyses carried out in this investigation determined that the control group and the experimental group were equivalent. This fact allowed us to develop the investigation in an adequate way.

The study was carried out through a seminar organized and authorized by the Provincial Directorate of Education of Ceuta, dependent on the Ministry of Education and Vocational Training. In addition, it has the approval of the directors of the educational centers involved in the study, as established in art. 120.4 on the autonomy of centers in Organic Law 2/2006, of 3 May, on Education in Spain [78]. The educational centers have the authorization of the families. The students carried out the study voluntarily and anonymously, and the data processing guaranteed data protection.

### 2.2. Instrument

For the research, a validated co-measure questionnaire was used based on the development of an evaluation of student collaboration in STEAM activities [46], located after a thorough review of the scientific literature.

This questionnaire is made up of a total of fifteen items distributed in five dimensions: (1) Interactions between peers (5 items), (2) Positive communication (3 items), (3) Rich consultation/Multiple pathways (2 items), (4) Authentic Approaches/Tasks (3 items) and (5) Transdisciplinary Thinking (2 items). The different variables were evaluated using a 3-point Likert rating scale (1—needs work, 2—acceptable and 3—proficient).

The validated instrument used is based on the US context and a K-12 sample [46]. In our case, based on the levels of validation and reliability of the questionnaire, we carried out a validation and reliability of the said instrument in the Spanish context, specifically for students in the sixth grade of primary education (Appendix A). In this case, the instrument was translated by two expert translators. Subsequently, a quantitative evaluation was carried out on the original instrument by five experts in the field of STEAM education in Spain. For this, the Fleiss Kappa and Kendall’s W tests were performed, which were correct (K = 0.81; W = 0.85) [79].

An exploratory factorial analysis was performed using the quantitative validation method through the main components of the method with a varimax rotation. The dependence between the variables was obtained with the sphericity test (Bartlett = 4221.043; df = 595, *p* < 0.0001). Additionally, a relevant score was discovered through the Kaiser–Meyer–Olkin test (KMO = 0.811). Finally, the mean reliability of the questionnaire was calculated with various statistical processes, such as the mean variance extracted (AVE = 0.78), Cronbach’s alpha (α = 0.84) and composite reliability (FC = 0.81). The results of these values confirm adequate levels of reliability of the instrument used.

### 2.3. Procedure

This study was approved by the ethics committee under code 2292/CEIH/2021, in accordance with the recommendations contained in the Declaration of Helsinki on good research practices. In addition, the study in the educational centers was carried out through a seminar organized and authorized by the Provincial Directorate of Education of Ceuta, dependent on the Ministry of Education and Vocational Training. It also has the approval of the directors of the educational centers involved in the study, as established in art. 120.4 on the autonomy of centers [78] on Education in Spain. The educational centers have the authorization of the families. The students carried out the study voluntarily and anonymously, and the data processing guaranteed data protection.

The research phase was carried out after the confinement caused by the first wave of COVID-19, specifically after the incorporation of students in face-to-face classes.

In the study phase, coinciding with the first week of incorporation of the students after the period of confinement, the researchers proceeded to collect data from the printed pre-test questionnaire that was provided to the students of sixth level of primary education, anonymously and voluntarily. This questionnaire referred to how they worked in face-to-face classes before the COVID-19 pandemic, since the students did not know how the classes would develop with the new normality established by the restrictions and sanitary measures. The researchers were present at the data collection and advised the students at all times.

In the experimental phase, the students of both groups, control and experimental, used the methodologies of their educational plans in person but taking into account the preventive measures mentioned in the justification and dictated by the health and educational legislation in Spain at that time [69]. After a quarter of classes, the researchers repeated the same procedure as the pre-test; this time, it was about collecting data from the post-test sample in relation to the methodology used during that quarter with the sanitary measures and restrictions. In this process, again, the researchers were present in the data collection and advised the students at all times. Finally, once the data were collected, they were transferred to the database of the Statistical Package for the Social Sciences (SPSS*_V.25_*). Once the said database was configured, its statistical analysis was carried out.

**Figure 4:**
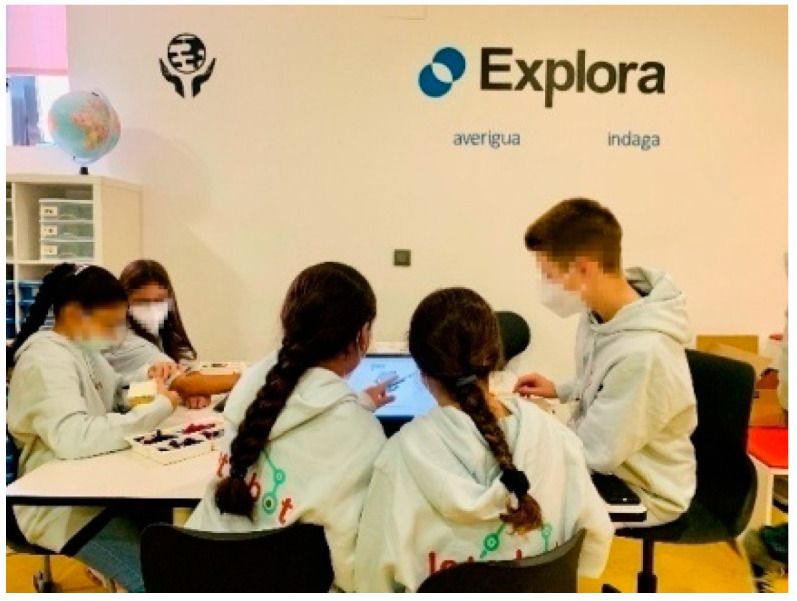
Future classroom.

The pedagogical method developed by the experimental group is framed within the Educational Project of the Center. This group has a methodological line for the entire primary stage, from 6 to 12 years old. Therefore, this group of sixth grade of primary education had worked on computational thinking and in makerspaces from an early age. This methodological line includes four content blocks: the computer and its operation, the internet, audiovisual image editing and computational thinking. In addition, it has the spaces that the European Schoolnet in Brussels and the INTEF advise to promote active methodologies (Figure 4). These zones are: Present, Create, Investigate, Interact, Develop and Explore. This way of working, alternating with the traditional methodology, that is, with the textbook as a guide for the teacher and students, was the work dynamics of the experimental group. Until the arrival of COVID-19 and the return to face-to-face classes, the restrictions only allowed the use of the traditional methodology, and these makerspaces could not be used.

### 2.4. Analysis of Data

To obtain the study data and respond to the objectives, an in-depth analysis was carried out. In the statistical analysis of the research, the mean (M), the standard deviation (SD) and the standard error of the mean (SE) were included. In addition, skewness (Skw) and kurtosis (Kme) tests were included to identify the distribution trend. The coefficient of variation (CV) was also used to find out the dispersion of the response.

These statistics were applied to identify whether or not it was feasible to use the Student’s t-test. The Student’s t-test (*t_n_*_1*+n*2*−*2_) was used to compare the means between the different groups. Additionally, to diagnose the size of the effect achieved after the training process, Cohen’s d and the biserial correlation (*r_xy_*) were used. The analysis was used, taking as reference the values of *p* < 0.05 as statistically significant differences.

All values of asymmetry and kurtosis were in the range of ± 1.95, following the premises established by Ref [80] for a normal distribution. The Student’s t-test statistic was measured by the degree of independence of the results obtained, although from two different perspectives. Independent samples were analyzed, that is, a comparison was made between the traditional teaching method and the groups that used active methodologies and makerspaces, both in the pre-test and in the post-test.

## 3. Results

The results obtained after applying the different statistical methods are presented below. In general and descriptively, in Table 1, the results of the control and experimental groups can be observed in the pre-test and post-test questionnaire.

In relation to the results of the pre-test data, both the experimental group and the control group presented similar values in each of the STEAM dimensions. For the control group, the dimension with the highest score with a mean of 2.30 was Positive Communication (POST_COM), and the one with the lowest score with a mean of 2.21 was Transdisciplinary Thinking (TRANS_THINK). For the experimental group in the pre-test, the dimension with the highest value was also POST_COM but with a mean greater than 2.35, in reference to the control group. In contrast, the least valued dimension with a mean of 2.18 was Authentic Approach and Tasks (AA_TASK).

In the analysis of the results of the post-test data, higher values were observed in all the study dimensions in the control group. Specifically, the most valued dimension in the experimental group taking the post-test was, again, POST_COM, with a mean of 2.63, that is, 0.33 points more than in the pre-test results. In contrast, the lowest valued dimension in the control group was Peer Interaction (PEER_INT), with a mean of 2.37. In turn, in the experimental group, the dimension with the highest mean value in the post-test results was also POST_COM, with a mean of 2.26, that is, 0.09 points less in relation to the pre-test results. On the other hand, the mean with the lowest score was the dimension PEER_INT 1.77, that is, 0.44 points less in relation to the pre-test results.

Considering the standard deviation, it was well below one point in all the study dimensions, both in the pre-test and post-test measures. In this case, it can be considered that there was no response dispersion. Students tended to agree on their answer. All asymmetry and kurtosis values were between +/−1.96, thus being the premises set by Ref [80] for a normal distribution. Finally, the kurtosis values showed that the response trend was mainly platykurtic.

**Figure 5:**
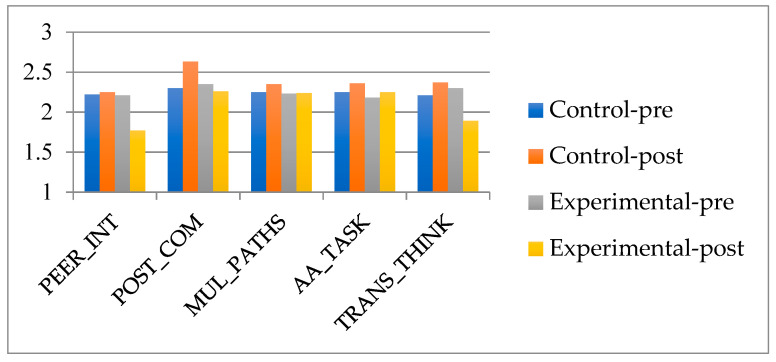
Comparison between control group and experimental group pre-test and post-test.

The comparison of means of the different study dimensions shows, graphically, in Figure 5, the following:

The POST_COM dimension is the most valued dimension in all the measurements taken, both in the control group and in the experimental group of the pre-test and post-test measures. On the other hand, the least valued dimension in each of the measures taken in each of the groups varies.

The great difference in the mean of the measurements of the experimental group stands out in the post-test results of the PEER_INT and TRANS_THINK dimensions, in relation to the rest of the dimensions and measurements. In these two cases, they usually have a difference of at least 0.34 points with respect to the rest of the dimensions. Both dimensions, in the pre-test results, have a higher value in the experimental group.

The degree of independence of the results obtained was introduced with the Student’s t-statistic, although from two different perspectives. On the one hand, independent samples were analyzed, that is, a comparison was made between the control groups and the experimental groups, both in the pre-test and post-test tests. The data visible in Table 2 show that the comparison between the control group and the experimental group is significant in the post-test measurement of the PEER_INT, POST_COM and TRANS_THINK dimensions. This shows significant differences in favor of the control group. In the rest of the dimensions and comparisons, no significant differences are observed. In the previously indicated dimensions, in which a significant relationship is observed, the biserial correlation is medium. The effect size is very low in all dimensions, both in those with a significant relationship and in those without a significant relationship.

On the other hand, the related samples were analyzed, that is, between the pre-test and post-test tests of the control and experimental groups (Table 3). In the experimental group, two dimensions are observed in which there are significant differences. This is the case for the PEER_INT and TRANS_THINK dimensions. In this case, the pre-test measures are superior to the post-test measures. That is, the evaluation of the students drops significantly in the experimental group. In the control group, there are significant differences in the pre-test and post-test measures in the COM_POS dimension. In this case, the post-test scores are higher than those of the pre-test. In the rest of the dimensions and comparisons, no significant differences are observed.

## 4. Discussion

After the suspension of classes in educational centers caused by the confinement of COVID-19, Spanish schools had to adapt spaces and resources to the demands and measures of political institutions in matters of education and health, as indicated by the government of Spain [69]. Educational centers have coexisted with technology, as Ref [1] points out, although this fact has presented a problem for schools in their educational projects in general and classroom methodologies in particular. In schools that include the STEAM methodology and have makerspaces and Classrooms of the Future in their teaching–learning process, it has meant a “before” and “after” the pandemic. In this sense, the axis of this STEAM-EDU study also suffered an impact on its disciplines for the students that we proceed to discuss.

### 4.1. In Relation to the First Study Objective. To Compare How Working with Steam-Edu Influences Each of the Dimensions of the Study before the Pandemic Using Different Methodologies

The data from the pre-test study showed different results in the control and experimental groups. We agree with Refs [21,22,23,33,34,40,41] that TRANS_THINK had a better statistical evaluation in this study among the students of the experimental group that used STEAM and computational thinking as a discipline that allows them to solve and approach a problem, not necessarily math, and work cooperatively in person. This fact confirms that discussing how to approach a task, activity or problem and co-creating projects across multiple disciplines contribute to the development of transversal and critical thinking. In addition, the lines of research of Refs [4,5,9,29,46,58,59] support the results of the experimental group that developed the creativity of students by exchanging opinions, ideas and carrying out co-creation of projects. It also allows one to check what other colleagues do when presenting their projects from any area of the curriculum, such as the “present area” within a Classroom of the Future, all from motivating work spaces for students.

Another discipline with the highest score for both groups, experimental and control, was POST_COM. However, there was a higher assessment in the experimental group over the control group. Therefore, these results support previous studies [2,33,36,66] where STEAM-EDU facilitates communication in schools, respecting the ideas of others, listening and respecting turn taking. In addition, these skills allow socially appropriate behavior among the peers of the students in their lives and develop oral expression and comprehension, contributing to linguistic competence, supporting the lines with the research of Refs [24,25] from the makerspaces and Classroom of the Future, as also pointed out by previous studies by Refs [58,59].

On the contrary, the discipline with the lowest value within the experimental group, although above the average, was Approaches and Tasks (AA_TASK), also with a lower value compared to the control group, which presented a higher value. These data are not in line with the results of previous studies by Refs [11,38,60]. Before the pandemic, the experimental group did not consider it relevant to use technological tools in collaboration to tackle tasks, reflective learning (STREAM), negotiate relevant methods or materials to solve a problem. Learning through online educational platforms individually or in groups was not considered necessary by the students. Therefore, the results of this discipline agree with the study by Ref [3], in that a change in methodological approaches was not necessary to achieve STEAM skills, in this discipline specifically and before the pandemic.

The data for the Peer Interaction (PEER_INT) and Multiple Options Consultation (MUL_PATHS) dimensions are above the mean of the study scale in both groups and with similar statistical data. These results support the lines of research of Refs [31,53,54,55] that highlight the lack of innovation in schools to develop disciplines, such as supervising and distributing tasks with their peers, negotiating roles, checking processes, asking appropriate questions to solve problems or verify research sources through STEAM methodologies in makerspaces and Classrooms of the Future, as established by previous studies by Refs [55,66].

The discussion of this objective responds positively with hypothesis H_1_, despite being a STEAM-EDU field with little research on methodologies and teaching resources, as pointed out [19,20,51]. The experimental group that developed the STEAM disciplines through computational thinking in makerspaces and Classroom of the Future had a significantly better assessment by the experimental group in two disciplines, TRANS_THINK and POST_COM. In addition, in two other disciplines, PEER_INT and MUL_PATHS, although the assessment was very similar, they were data that we can consider positive. By using a STEAM methodology, these disciplines that require training among teachers and are in the process of being implemented in the official curricula of primary education in Spain, as also pointed out [2,56,57], at least do not have a worse evaluation between students in the experimental group versus the control group.

### 4.2. In Relation to the Second Objective of the Study. To Know the Impact That the COVID-19 Pandemic Has Had on the STEAM Disciplines

Before the pandemic, the STEAM-EDU methodology, through computational thinking from collaborative and creative learning spaces, had a worse rating in the AA_TASK dimension. However, it is noteworthy that the only dimension that experienced a significantly better assessment in the experimental group in the post-test with reference to the pre-test was AA_TASK. In line with the study of Ref [11], the pandemic, the confinement, and later, the restrictions in the classroom made the students of the experimental group of this research better value the need to work with online digital platforms.

In line with Ref [67] and in accordance with the results of the experimental group of this study, the POST_COM and TRANS_THINK disciplines improved in the Classroom of the Future. However, unlike the first objective that we discussed, the data confirm that the pandemic, together with the preventive measures in Spanish schools, had an impact on the experimental group that developed active methodologies, as supported by the study by Ref [68], especially in PEER_INT, POST_COM and TRANS_THINK in a negative way, although above the average evaluation. That is, working in the classroom as a group or exchanging ideas among peers through fields of computational thinking, such as robotics, programing or artificial intelligence, as supported by studies by Refs [41,42,43,51], are considered beneficial for transdisciplinary learning. However, according to the results of this study, the students of the experimental group valued these disciplines less when working individually and without being able to share resources or being able to move to creative spaces.

The results of this research linked to the PEER_INT dimension and its variables, in relation to previous studies by Refs [21,22], which support carrying out face-to-face collaborative activities that require direct interaction between students, are not in line with the results of this study during the pandemic in the experimental group that developed STEAM-EDU methodologies due to the impact of restrictions in the classroom for health reasons. In the same way, the studies by Refs [6,67] that support the use of creative spaces of makerspaces and the Classroom of the Future to investigate, interact, explore, develop, create and present were less valued by the experimental group in the post-test data.

The discussion of this objective nullifies hypothesis H_2_, that is, the STEAM-EDU disciplines during the pandemic are better valued in the experimental group compared to the control group. Although, in reality, the post-test results are evidently lower in the experimental group than in the control, even in reference to the experimental group itself in the pre-test. We can consider these results in favor of STEAM-EDU when classes return to full normality and without restrictions. This is due to the fact that the STEAM-EDU methodology in spaces such as makerspaces and Aula del Futuro generates motivation, as indicated by previous studies [27,28], and the restrictions imposed by the pandemic have interrupted a way of working that the experimental group considered positive and effective.

In reference to the main objective of the study, “Evaluate the STEAM dimensions in sixth grade of primary education in times of pandemic”, the post-test results of the experimental group support the lines of research of Refs [19,20] in relation to the fact that there is little research on STEAM-EDU teaching methodologies and resources among teachers, who encountered an added difficulty during the pandemic, that is, the restrictions implemented in educational centers by the government of Spain [69]. Finally, in the experimental group, the possibility of creating, researching, interacting, exploring, developing and presenting, which are areas of the Classroom of the Future, and these actions are associated with the pyramid of Bloom’s taxonomy, are enriching cognitive processes that are achieved through computational thinking, following the lines of research in Refs [40,43,44]. In this study, the pre-test data show that they are viable but not as a result of the pandemic.

## 5. Conclusions

The conclusions of this research are that the methodologies based on computational thinking within makerspaces and Classroom of the Future before the pandemic were developing a positive impact on the STEAM disciplines, except in the AA_TASK dimension, where the use of digital platforms to work in the classroom was still not so relevant at that time. Therefore, the motivation generated by students in this digital society to work with technological tools and resources in the classroom is an unquestionable fact that has repercussions on STEAM disciplines, always from a didactic and pedagogical approach.

Another conclusion of this study is that the restrictions on the use of common spaces and the exchange of technological resources due to the pandemic have been detrimental to educational centers that develop computational thinking in creative spaces, harming STEAM disciplines because this methodology implies “know-how” and a bidirectional education, where the teacher is the guide and the students the protagonists. However, we can verify that a drop in the ratio after the pandemic positively influenced the evaluations of the control group in the post-test, not being able to determine what would have happened if the experimental group had worked on active methodologies through computational thinking in makerspaces and Classrooms of the Future in smaller groups without health restrictions.

Among the main limitations of this study is the sample selected only for students in the sixth year of primary education and in a specific sociodemographic area in Spain. This study can be transposed to other educational levels and stages and related to other variables, such as gender, sociodemographic characteristics or the use of electronic devices in homes and schools.

As future lines of research, studies can be carried out from September 2022 on the impact that STEAM disciplines will have on Spanish schools. The government of Spain will implement the School Code Plan 4.0 and will involve an investment of EUR 356 million over the next two years, officially including computational thinking and robotics in its curriculum in the stages of early childhood education, primary education and secondary education in centers supported by public funds. These fields are considered by the Spanish government as the new language of the present and future in schools.

The findings of this study are of interest to researchers and professionals in the educational world, both in formal and informal education. It is important to be aware that technological and digital tools and devices are prominent in many daily tasks, including in the world of work. Policies and centers must include training activities related to computational thinking from makerspaces and Classrooms of the Future from an early age to promote STEAM disciplines. If the world of work needs professions that require digital and technological skills, schools are the first link to achieve it.

## Figures and Tables

**Table 1:** Results obtained for the dimensions of study in GC and GC of secondary education.

	Parameters								
	Dimensions	Pre-Test				Post-Test			
		M	SD	S_kw_	K_me_	M	SD	S_kw_	K_me_
**Control** **group**	INT_PARES	2.22	0.463	−0.335	−0.736	2.25	0.447	−0.137	−0.832
	COM_POS	2.30	0.444	0.158	−0.713	2.63	0.436	−1.22	0.956
	INV_RES_PRO	2.25	0.592	−0.600	−0.333	2.35	0.460	−0.224	−0.718
	ENF_TAR	2.25	0.585	−0.313	−0.518	2.36	0.504	−0.685	−0.017
	PEN_TRANS	2.21	0.524	−0.518	0.121	2.37	0.489	−0.320	−0.843
**Experimental group**	INT_PARES	2.21	0.502	−0.367	−0.498	1.77	0.515	0.605	−0.175
	COM_POS	2.35	0.414	−0.445	−0.608	2.26	0.438	−0.200	−0.106
	INV_RES_PRO	2.23	0.519	−0.369	−0.016	2.24	0.513	−0.648	0.105
	ENF_TAR	2.18	0.537	−0.456	−0.456	2.25	0.567	−0.440	−0.744
	PEN_TRANS	2.30	0.516	−0.514	−0.014	1.89	0.654	0.146	−1.70

**Table 2:** Study of the value of independence between independent samples with pre-test and post-test. Student’s t-test for independent samples.

Dimensions		µ(X_1_–X_2_)	*t_n_*_1+*n*2−2_	df	*d*	r_xy_
PEER_INT	pre	(2.22–2.21)	0.085	122	−0.013	−0.008
	pos	(2.25–1.77)	5.241 **	122	−0.046	−0.429
POST_COM	pre	(2.30–2.35)	−0.577	122	−0.043	0.052
	pos	(2.63–2.26)	4.514 **	122	0.041	−0.378
MUL_PATHS	pre	(2.25–2.23)	0.195	122	0.054	−0.018
	pos	(2.35–2.24)	1.217	122	−0.020	−0.110
AA_TASK	pre	(2.25–2.18)	0.599	122	−0.029	−0.054
	pos	(2.36–2.25)	1.020	122	0.007	−0.092
TRANS_THINK	pre	(2.21–2.30)	−0.944	122	−0.005	0.085
	pos	(2.37–1.89)	4.367 **	122	−0.063	−0.368

**Note:** µ = Mean difference; X_1_ = control group; X_2_ = experimental group; ** Correlation is significant at the 0.01 level; n.s. Correlation not significant.

**Table 3:** Study of the value of independence between dependent samples between control group and experimental group. Student’s t-test for related samples.

Dimensions		µ(Y_1_–Y_2_)	*t_n_*_1*+n*2−2_	df	SD	SEA
PEER_INT	con	−0.029 (2.22–2.25)	−0.375	47	0.539	0.077
	exp	0.436 (2.21–1.77)	5.505 **	75	0.691	0.079
POST_COM	con	−0.326 (2.30–2.63)	−4.264 **	47	0.530	0.076
	exp	0.083 (2.35–2.26)	1.572	75	0.462	0.053
MUL_PATHS	con	−0.104 (2.25–2.35)	−1.032	47	0.699	0.101
	exp	−0.013 (2.23–2.24)	−0.188	75	0.610	0.070
AA_TASK	con	−0.111 (2.25–2.36)	−1.141	47	0.674	0.097
	exp	−0.070 (2.18–2.25)	−1.056	75	0.579	0.066
TRANS_THINK	con	−0.156 (2.21–2.37)	−1.475	47	0.620	0.089
	Exp	0.414 (2.30–1.89)	4.875 **	75	0.741	0.085

**Note:** µ = Mean difference; Y_1_ = pre-test; Y_2_ = post-test; ** Correlation is significant at the 0.01 level; n.s. Correlation not significant.

## Data Availability

Data collected for this study can be made available upon request.

## List of Abbreviations

**STEM**	Science, Technology, Engineering and Mathematics
**STEAM**	Science, Technology, Engineering, Art and Mathematics
**STEAM-EDU**	Science, Technology, Engineering, Art and Mathematics in Education
**INTEF**	National Institute of Educational Technology and Teacher Training
**ICT**	Information and Communication Technology
**PEER_INT**	Peer Interaction
**POST_COM**	Positive Communication
**MUL_PATHS**	Multiple Paths
**AA_TASK**	Authentic Approach and Tasks
**TRANS_THINK**	Transdisciplinary Thinking

## Appendix A

**Table A1:** Questionnaire. Co-Measure: developing an assessment for student collaboration in STEAM activities.

**1. Dimension: Peer Interaction (PEER_INT)**
Monitors tasks/project with peers; Negotiates roles within group; Divides and works toward task completion; Checks for understanding regarding process and/or content; Provides peer feedback, assistance and/or redirection.
**2. Dimension: Positive Communication (POST_COM)**
Respects others’ ideas; Uses socially appropriate language and behavior; Listens and takes turns.
**3. Dimension: Multiple Paths (MUL_PATHS)**
Develops appropriate questions toward solving the problem; Verifies information and sources to support inquiry.
**4. Dimension: Authentic Approach and Tasks (AA_TASK)**
Shares connections to relevant knowledge; Negotiates method or materials relevant to solving the problem posed; Uses tools collaboratively to approach task.
**5. Dimension: Transdisciplinary Thinking (TRANS_THINK)**
Discusses approaching task, activity or problem using multiple disciplines; Co-creates products by incorporating multiple disciplines.

## References

[1] Choi I. (2022). A Study on Spatial Characteristics of Flipped Learning-based STEAM Educational Environment. J. Korea Inst. Spat. Des..

[2] Chan P., Hyun J. (2022). Review on Teachers’ Digital Competency Based on Digital Technology Integration Model for 2022 Revised Curriculum. J. Korean Assoc. Comput. Educ..

[3] Vargas J., Cuero J., Riveros F. (2020). Digital transformation and STEAM approach, an alternative in times of COVID-19. J. Spaces.

[4] Casado R., Checa M. (2020). Robotics and STEAM Projects: Development of creativity in Primary Education classrooms. Píxel-Bit Rev. Medios Educ..

[5] Ruíz F., Zapatera A., Montes N. (2020). Curriculum analysis and design, implementation, and validation of a STEAM project through educational robotics in primary education. Comput. Appl. Eng. Educ..

[6] Sánchez E. (2019). STEAM education and the “maker” culture. J. Parents Teach..

[7] Toma R.B., Greca I.M. (2018). The effect of integrative STEM instruction on elementary students’ attitudes toward science. Eurasia J. Math. Sci. Technol. Educ..

[8] Kim J.O., Kim J. (2018). Development and Application of Art Based STEAM Education Program Using Educational Robot. Int. J. Mob. Blended Learn..

[9] Conradty C., Bogner F.X. (2020). How Creativity in STEAM Modules Intervenes with Self-Efficacy and Motivation. Educ. Sci..

[10] Santillán J., Vaca V., Santos R., Jaramillo E. (2020). Steam Methodology, as a Resource for Learning in Higher Education. INTED2020, Proceedings of the 14th International Technology, Education and Development Conference, Valencia, Spain, 2–4 March 2020.

[11] Makrakis V. From STEM to STEAM and to STREAM enabled through meaningful critical reflective learning. Proceedings of the 2nd International Conference on Innovating STEM Education.

[12] Marín J.A., Moreno A.J., Dúo P., López J. (2021). STEAM in education: A bibliometric analysis of performance and co-words in Web of Science. Int. J. STEM Educ..

[13] Ozkan G., Umdu U. (2021). Exploring the effectiveness of STEAM design processes on high school students’ creativity. Int. J. Technol. Des. Educ..

[14] Colucci L., Burnard P., Gray D., Cooke C. A Critical Review of STEAM (Science, Technology, Engineering, Arts and Mathematics). https://oxfordre.com/education/view/10.1093/acrefore/9780190264093.001.0001/acrefore-9780190264093-e-398.

[15] Xue H. (2022). A New Integrated Teaching Mode for Labor Education Course Based on STEAM Education. Int. J. Emerg. Technol. Learn..

[16] Anisimova T., Sabirova F., Shatunova O. (2020). Formation of Design and Research Competencies in Future Teachers in the Framework of STEAM Education. Int. J. Emerg. Technol. Learn..

[17] Anito J., Morales M.P. (2019). The Pedagogical Model of Philippine STEAM Education: Drawing Implications for the Reengineering of Philippine STEAM Learning Ecosystem. Univ. J. Educ. Res..

[18] Nepeina K., Istomina N., Bykova O. (2020). The Role of Field Training in STEM Education: Theoretical and Practical Limitations of Scalability. Eur. J. Investig. Health Psychol. Educ..

[19] Greca I., Ortiz J., Arriassecq I. (2021). Design and evaluation of a STEAM teaching-learning sequence for primary education. J. Eureka Teach. Dissem. Sci..

[20] Hong J.C., Ye J.H., Ho Y.J., Ho H.Y. (2020). Developing inquiry and hands-on learning model to guide STEAM lesson planning for kindergarten children. J. Balt. Sci. Educ..

[21] De la Garza A. (2019). Internationalizing the Curriculum for STEAM (STEM + Arts and Humanities): From Intercultural Competence to Cultural Humility. J. Stud. Int. Educ..

[22] Gao X., Li P., Shen J., Sun H. (2020). Reviewing assessment of student learning in interdisciplinary STEM education. Int. J. STEM Educ..

[23] Staribratov I., Manolova N. (2022). Application of mathematical models in graphic design. Math. Inform..

[24] Kazakoff E., Sullivan A., Bers M. (2012). The Effect of a Classroom-Based Intensive Robotics and Programming Workshop on Sequencing Ability in Early Childhood. Early Child. Educ. J..

[25] Duo P., Hinojo F.J., Moreno A.J., López J.A. (2022). STEAM in Primary Education. Impact on Linguistic and Mathematical Competences in a Disadvantaged Context. Front. Educ..

[26] Dúo P., Moreno A.J., Marín J.A. (2022). ICT Motivation in Sixth-Grade Students in Pandemic Times. The Influence of Gender and Age. Educ. Sci..

[27] Huang F. (2020). Effects of the Application of STEAM Education on Students’ Learning Attitude and Outcome. The Case of Fujian Chuanzheng Communications College. Rev. Cercet. Interv. Soc..

[28] Elmi C. (2020). Integrating Social Emotional Learning Strategies in Higher Education. Eur. J. Investig. Health Psychol. Educ..

[29] Davies R., Trowsdaleb J. (2021). The culture of disciplines: Reconceptualising multi-subject curricula. Eur. J. Investig. Health Psychol. Educ..

[30] Pahmi S., Juandi D., Sugiarn R. (2022). The Effect of STEAM in Mathematics Learning on 21st Century Skills: A Systematic Literature Reviews. PRISMA.

[31] Chung S.K., Li D. (2021). Issues-Based STEAM education: A case study in a Hong Kong secondary school. Int. J. Educ. Arts.

[32] Chan P., Hyun J. (2020). K-TIHM: Korean Technology Integration Hierarchy Model for Teaching and Learning in STEAM Education. J. Korean Soc. Inf. Technol..

[33] Chang D., Hwang G.J., Chang S.C., Shen Y. (2021). Promoting students’ cross-disciplinary performance and higher order thinking: A peer assessment-facilitated STEM approach in a mathematics course. Educ. Technol. Res. Dev..

[34] Urgiles B.E., Tixi K.G., Allauca M.E. (2022). STEAM methodology in academic environments. Dominio Cienc..

[35] Dubek M., DeLuca C., Rickey N. (2021). Unlocking the potential of STEAM education: How exemplary teachers navigate assessment challenges. J. Educ. Res..

[36] Al-Mutawah M.A., Thomas R., Preji N., Alghazo Y.M., Mahmoud E.Y. (2022). Theoretical and Conceptual Framework for A STEAM-Based Integrated Curriculum. J. Posit. Sch. Psychol..

[37] Subramaniam V., Karpudewan M., Roth W.M. (2022). Unveiling the Teachers’ Perceived Self-efficacy to Practice Integrated STrEaM Teaching. Asia-Pac. Educ. Res..

[38] Lim S., Tan K. (2022). Teaching Descriptive Writing via Google Classroom Stream: Perception Among Year 6 Primary Students. Theory Pract. Lang. Stud..

[39] Bati K., Yetişir M., Çalişkan I., Gunes G., Saçan E. (2018). Teaching the concept of time: A steam-based program on computational thinking in science education. Cogent Educ..

[40] Moreno J., Robles G., Román M., Rodríguez J.D. (2019). Not the same: A text network analysis on computational thinking definitions to study its relationship with computer programming. RIITE. Interuniv. J. Res. Educ. Technol..

[41] González M.O., González Y.A., Muñoz C. (2021). Panorama of educational robotics in favor of STEAM learning. Eureka J. Sci. Teach. Dissem..

[42] Tengler K., Kastner O., Sabitzer B., Lavicza Z. (2022). The Effect of Robotics-Based Storytelling Activities on Primary School Students’ Computational Thinking. Educ. Sci..

[43] Su Y.-S., Shao M., Zhao L. (2022). Effect of Mind Mapping on Creative Thinking of Children in Scratch Visual Programming Education. J. Educ. Comput. Res..

[44] Rodríguez J., Moreno J., Román M., Robles G. (2021). Evaluation of an Online Intervention to Teach Artificial Intelligence with LearningML to 10–16-Year-Old Students. Proceedings of the 52nd ACM Technical Symposium on Computer Science Education (SIGCSE ’21).

[45] Conradty C., Bogner F.X. (2020). STEAM teaching professional development works: Effects on students’ creativity and motivation. Smart Learn. Environ..

[46] Herro D., Quigley C., Andrews J., Delacruz G. (2017). Co-Measure: Developing an assessment for student collaboration in STEAM activities. Int. J. STEM Educ..

[47] Álvarez M. (2017). Development of computational thinking in primary education: An educational experience with Scratch. J. Educ. Sci..

[48] Roig R., Moreno V. (2020). Computational thinking in Education. Bibliometric and thematic analysis. Rev. Educ. Distancia.

[49] (2022). Royal Decree 157/2022, of 1 March, Which Establishes the Organization and Minimum Teachings of Primary Education—State Official Bulletin No. 52.

[50] Yunusova G. (2022). Programming and robotics based in steam learning. Am. J. Interdiscip. Res. Dev..

[51] Rodríguez J.D., Moreno J., Román M., Robles G. (2020). LearningML: A Tool to Foster Computational Thinking Skills through Hands-on Artificial Intelligence Projects. Rev. Educ. Distancia.

[52] Dúo P., López L., Pozo S., Carmona N., Carrión J.J., López L., Reyes M., Pérez E. (2022). Computational thinking in education. Didactic Proposals and Research in the Higher Education.

[53] Chondrogiannis E., Symeonaki E., Papachristos D., Loukatos D., Arvanitis K.G. (2021). Computational Thinking and STEM in Agriculture Vocational Training: A Case Study in a Greek Vocational Education Institution. Eur. J. Investig. Health Psychol. Educ..

[54] Tenhovirta S., Korhonen T., Seitamaa-Hakkarainen P., Hakkarainen K. (2022). Cross-age peer tutoring in a technology-enhanced STEAM project at a lower secondary school. Int. J. Technol. Des. Educ..

[55] Herro D., Quigley C., Abimbade O. (2021). Assessing elementary students’ collaborative problem-solving in makerspace activities. Int. J. Technol. Des. Educ..

[56] Juskeviciene A. (2020). STEAM Teacher for a Day: A Case Study of Teachers’ Perspectives on Computational Thinking. Inform. Educ..

[57] Webb D., LoFaro K. (2020). Sources of engineering teaching self-efficacy in a STEAM methods course for elementary preservice teachers. Sch. Sci. Math..

[58] Forte J., Ibarra L., Glasserman L.D. (2021). Analysis of Creative Thinking Skills Development under Active Learning Strategies. Educ. Sci..

[59] Freundt V. (2019). The Makerspace as a Space to Foster Creativity and Collaborative Learning in 4th and 5th Grade High School Students from a Public School in Callao from a Formal Educational Approach. Master’s Thesis.

[60] Kumar A., Archana G.S., Deepti K. (2022). Impact of AR-based collaborative learning approach on knowledge gain of engineering students in embedded system course. Educ. Inf. Technol..

[61] Ramos M., Cáceres M.P., Soler R., Marín J.A. (2020). The use of ICTs to encourage reading in vulnerable contexts: A systematic review in the last decade. Texto Livre Belo Horiz.-MG.

[62] Vázquez E., Gómez J., Infante A., López E. (2020). Incidence of a Non-Sustainability Use of Technology on Students’ Reading Performance in Pisa. Sustainability.

[63] Suarez A., García D., Martínez P., Torres J. (2018). Contribution of educational robotics in the acquisition of mathematical knowledge in primary education/contribution of educational robotics in the acquisition of mathematical knowledge in primary education. Magister.

[64] Tena R., Carrera N. (2020). The future classroom lab as a framework for the development of competency-based learning and project work. Mex. J. Educ. Res..

[65] INTEF (2022). Future Classroom.

[66] Sucheta K. (2022). Effectiveness of ‘STREAM based Learning Approach’ on Achievement in Science of Elementary School Students. Int. J. Innov. Sci. Res. Technol..

[67] Peña B., Caballero M.E., Mateo F. Technological Innovation Project Application of the Classroom of the Future. https://www.academia.edu/55832732/Innovaci%C3%B3n_tecnol%C3%B3gica_aplicando_el_Aula_del_Futuro.

[68] Román F., Fores A., Calandri I., Gautreaux R., Antúnez A., Ordehi D., Calle L., Poenitz V., Correa K.L., Torresi S. (2020). Resilience of teachers in mandatory preventive social distancing during the COVID-19 pandemic. J. Neuroeducation.

[69] Government of Spain (2020). Prevention, Hygiene and Health Promotion Measures against COVID-19 for Educational Centers in the 2020–2021 Academic Year.

[70] Corral D., Fernández J.J. (2021). Education exposed after the pandemic of COVID-19. Shortcomings and challenges. Aularia.

[71] Mailizar A., Maulina S., Bruce S. (2020). Secondary School Mathematics Teachers’ Views on E-learning Implementation Barriers during the COVID-19 Pandemic: The Case of Indonesia. Eurasia J. Math. Sci. Technol. Educ..

[72] García R.O., García C.E. (2020). STEAM methodology and its use in Mathematics for high school students in times of the COVID-19 pandemic. Sci. Domain.

[73] Hernández R., Fernández C., Baptista P., Hernández R., Fernández C., Baptista P. (2014). Definition of the scope of the research to be carried out: Exploratory, descriptive, correlational or explanatory. Investigation Methodology.

[74] López J.A., López J., Moreno A.J., Marín J.A. (2020). Dietary Intervention through Flipped Learning as a Techno Pedagogy for the Promotion of Healthy Eating in Secondary Education. Int. J. Environ. Res. Public Health.

[75] Marín J.A., Soler R., Moreno A.J., López J. (2020). Effectiveness of Diet Habits and Active Life in Vocational Training for Higher Technician in Dietetics: Contrast between the Traditional Method and the Digital Resources. Nutrients.

[76] Fernández A. (2020). Social coexistence and linguistic challenges in Ceuta and Melilla. Hispanismes.

[77] INTEF (2022). Network of Future Classroom Centers.

[78] Organic Law 2/2006, of 3 May on Education—Official State Bulletin, No. 106, of 4 May 2006. https://www.boe.es/eli/es/lo/2006/05/03/2.

[79] Landis J.R., Koch G.G. (1977). The measure of observer agreement for categorical data. Biometría.

[80] Jöreskog K.G. (2001). Analysis of Ordinal Variables 2: Cross-Sectional Data—Text of the Workshop “Structural Equation Modelling with LISREL 8.51”.

